# Cannabidiol as a possible treatment for endometriosis through suppression of inflammation and angiogenesis

**DOI:** 10.1002/iid3.1370

**Published:** 2024-08-07

**Authors:** Roghayeh Anvari Aliabad, Kamyab Hassanpour, Amir Hossein Norooznezhad

**Affiliations:** ^1^ Department of Obstetrics and Gynecology Hamadan University of Medical Sciences Hamadan Iran; ^2^ School of Medicine, Hamadan University of Medical Sciences Hamadan Iran; ^3^ Medical Biology Research Center, Health Technology Institute Kermanshah University of Medical Sciences Kermanshah Iran

**Keywords:** angiogenesis, cannabidiol, cannabinoids, endometriosis, inflammation

## Abstract

**Background:**

Endometriosis is associated with a wide variety of signs and symptoms and can lead to infertility, embryo death, and even miscarriage. Although the exact pathogenesis and etiology of endometriosis is still unclear, it has been shown that it has a chronic inflammatory nature and angiogenesis is also involved in it.

**Objective:**

This review aims to explore the role of inflammation and angiogenesis in endometriosis and suggest a potential treatment targeting these pathways.

**Findings:**

Among the pro‐inflammatory cytokines, studies have shown solid roles for interleukin 1β (IL‐β), IL‐6, and tumor necrosis factor α (TNF‐α) in the pathogenesis of this condition. Other than inflammation, angiogenesis, the formation of new blood vessels from pre‐existing capillaries, is also involved in the pathogenesis of endometriosis. Among angiogenic factors, vascular endothelial growth factor (VEGF), hypoxia‐inducible factor 1α (HIF‐1α), transforming growth factor β1 (TGF‐β1), and matrix metalloproteinases (MMPs) are more essential in the pathogenesis of endometriosis. Interestingly, it has been shown that inflammation and angiogenesis share some similar pathways with each other that could be potentially targeted for treatment of diseases caused by these two processes. Cannabidiol (CBD) is a non‐psychoactive member of cannabinoids which has well‐known and notable anti‐inflammatory and antiangiogenic properties. This agent has been shown to decrease IL‐1β, IL‐6, TNF‐α, VEGF, TGFβ, and MMPs in different animal models of diseases.

**Conclusion:**

It seems that CBD could be a possible treatment for endometriosis due to its anti‐inflammatory and antiangiogenic activity, however, further studies are needed.

## Graphical abstract

Endometriosis is a chronic disease in which inflammation and angiogenesis are involved in it. Cannabidiol is an anti‐inflammatory and antiangiogenic agent derived from *Cannabis sativa*. It seems that cannabidiol could be an effective treatment for endometriosis through inhibition of inflammation and angiogenesis.

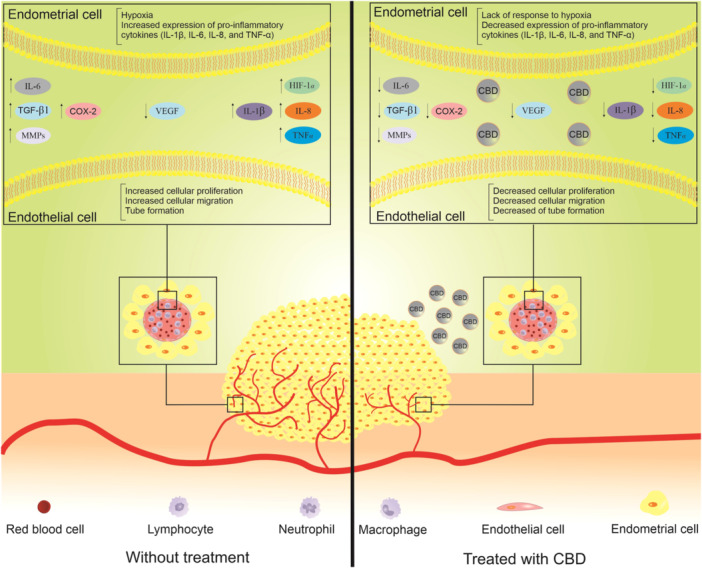

## 1 INTRODUCTION

Reports estimate the endometriosis prevalence to be 10%–15% in the general female population of reproductive age.^1^ This pathology is associated with dysmenorrhea, dyspareunia, dyschezia, and dysuria.^2^ Clinically, endometriosis could increase the risk of infertility,^3^ embryo death, and even miscarriage.^4^ Considering the mentioned outcomes, this condition strongly affects life quality and socioeconomically condition of those affected.^5^ Endometriosis is recognized by the existence of functional endometrial glands as well as stroma in exterior side of the uterine cavity in a chronic setting.^1^ Among the common sites of presence, ovaries, the uterosacral ligaments, the fossa ovarica, and the posterior cul‐de‐sac are more prevalent than the others.^1^ In some aspects, endometriosis is comparable with malignancies as they both tend to be recurrent, invasive, metastatic, and have estrogen‐dependent growth.^6^, ^7^ Although the exact etiology and mechanism of endometriosis is still unknown, retrograde menstruation is seriously suspected. However, other causes/etiologies such as immune system abnormalities, genetics and environmental issues, and coelomic metaplasia have been considered as well. It has been suggested that it's possible for at least two of the mentioned causes to be involved together.^2^ This article aims to review the role of inflammation and angiogenesis in the endometriosis and suggest a new possible treatment candidate for research: cannabidiol (CBD).

## 2 SEARCH STRATEGY

For this study, different databases such as PubMed, Scopus, and ISI Web of Science were searched from the inception to Jun 2023 with keywords including “Endometriosis; Angiogenesis; Inflammation; Cannabinoids; Cannabidiol.” Only English studies were included and different types of studies such as in vitro, ex vivo, in vivo, and clinical trials were used for data extraction.

### 2.1 Endometriosis and inflammation

Endometriosis is considered as a chronic inflammatory condition.^4^ The pro‐inflammatory cytokines such as interleukin 1β (IL‐β), IL‐6, and tumor necrosis factor α (TNF‐α) not only increase the inflammation but also are involved in the adhesion of endometrial tissue to the peritoneal surface. Also, they could increase production of metalloproteinases (MMPs) which leads to shedding of the endometrial tissue fragments.^8^

IL‐1β is a pro‐inflammatory cytokine from interleukin‐1 family which gets activated in the inflammasome to exert a wide range of inflammatory action.^9^ As it has been shown, levels of proIL‐1β and IL‐1β in the peritoneal fluid of women with endometriosis were significantly higher than controls.^10^, ^11^ As we showed before, the serum levels of IL‐1β were significantly higher in patients with endometrioma compared to healthy controls.^12^ Also, the levels of this pro‐inflammatory cytokine have been shown to be associated with the dyspareunia in the women with endometriosis.^13^

Inflammasomes are multi‐protein structures involved in both innate and adaptive immune system responses. They are involved in the production of different ILs such as IL1β. Aside from their IL production roles, inflammasomes are also key role players of endometriosis and, therefore, potent treatment targets.^14^ According to the investigations, estrogen receptor‐β (ERβ) is greatly increased in the endometriosis compared to normal endometrial tissues. It has been shown that ERβ of endometrial cells interacts with “cell death machinery” to prevent the TNF‐α‐induced apoptosis. Also, it increases IL‐1β production through an inflammasome‐mediated pathway to boost the cellular proliferation. Among the inflammasomes that ERβ interacts with in the endometriotic tissues are NOD‐, LRR, caspase I, and pyrin domain‐containing 3 (NLRP3) which all are involved in IL‐1β production pathway.^15^

IL‐6, an acute phase reactant, is a very crucial cytokine in inflammation and immune response.^16^ According to the results of our previous study, IL‐6 levels (serum) were significantly higher in patients with endometrioma compared to healthy controls.^12^ It has been shown that the expression of this cytokine was increased in the endometriotic tissues compared to the normal endometrial cells. Also, treatment of the mentioned cells with IL‐1β led to a dramatic increase in IL‐6 expression.^17^ Moreover, it has been shown that the expression of IL‐6 and soluble IL‐6 in plasma and peritoneal fluid was significantly higher in endometriotic patients compared to the controls.^18^ Also, there are other studies implicating the involvement of IL‐6 in the pathogenesis of endometriosis.^19^, ^20^

TNF‐α is a potent inflammatory cytokine of the TNF superfamily with a notable role in inflammation.^21^ This inflammatory cytokine has been shown to be involved in the pathogenesis of endometriosis.^4^ According to the investigations, TNF‐α is upregulated in the endometrial tissues of patients with endometriosis compared to normal endometrial tissues which supports the role of inflammation in this disorder.^2^ Also, the level of IL‐16 is found to be increased in the peritoneal fluid of cases diagnosed with endometriosis. This in turn suggests a possible involved pathway in the inflammatory process of this disorder.^22^ Not only IL‐16, but also IL‐8 and Regulated on Activation, Normal T Cell Expressed and Secreted (RANTES or CCL5) are increased in peritoneal fluid of women with endometriosis.^11^, ^23^ Moreover, peritoneal fluid analysis in the early stage endometriosis has revealed the increased levels of IL‐12.^24^

### 2.2 Endometriosis and angiogenesis

Angiogenesis is defined as new blood vessel formation from the pre‐existing capillaries. This process is observed in a few physiological conditions including female reproductive cycle^25^ and wound healing.^26^ On the other hand, angiogenesis participates in the pathogenesis of solid tumors growth,^27^ corneal neovascularization,^28^ hemophilic arthropathy,^29^ psoriasis,^30^ and endometriosis.^31^ Also, endometriosis is associated with a chronic inflammatory response^4^ that could cause endothelial dysfunction.^32^ Same as the tumors, angiogenesis and “dense vascularization” has been known as a characteristic condition in endometriosis for which antiangiogenesis therapies have been suggested in different studies.^31^, ^33^, ^34^

An increased unmet demand for oxygen leading to hypoxia could cause overexpression of hypoxia‐inducible factor 1α (HIF‐1α) which in turn could activate certain downstream pathways. The expression of HIF‐1α has been demonstrated to be significantly increased in ectopic endometriotic lesions compared to the controls.^35^, ^36^ Also, vascular endothelial growth factor (VEGF) and HIF‐1α are both proven to be overexpressed in the endometrial tissues with ectopic location.^36^, ^37^ In cases with ovarian endometriosis, an increased expression of HIF‐1α and nitric oxide synthase isoforms has been detected.^38^ In the ovarian endometriomas, the levels of VEGF and cyclooxygenase 2 (COX‐2) are found to be increased and correlate with each other, implicating the role of angiogenesis in ovarian endometriosis.^39^ In a systematic review and meta‐analysis on 13 published papers on animal models, anti‐VEGF/VEGF receptor therapy was found successful for treatment of endometriosis via suppression of angiogenesis.^40^ Also, it has been shown that transforming growth factor β1 (TGF‐β1) was increased in the peritoneum, peritoneal fluid, ectopic endometrium, and sera of women with endometriosis in compression to the controls.^41^ MMPs are among the important angiogenic factors that degrade basement membrane for facilitating the migration of endothelial cells. As mentioned above, these factors are extremely expressed in the endometriosis and the levels of their inhibitor follow a decreasing pattern.^42^ As described, MMPs are involved in the pathogenesis of endometriosis secondary to the inflammation. Studies have shown impaired or increased production of MMP‐1, MMP‐2, MMP‐3, and MMP‐9 as well as tissue inhibitors of MMP‐1 (TIMP‐1) and TIMP‐2.^42^

### 2.3 Inflammation and angiogenesis

**Figure 1:**
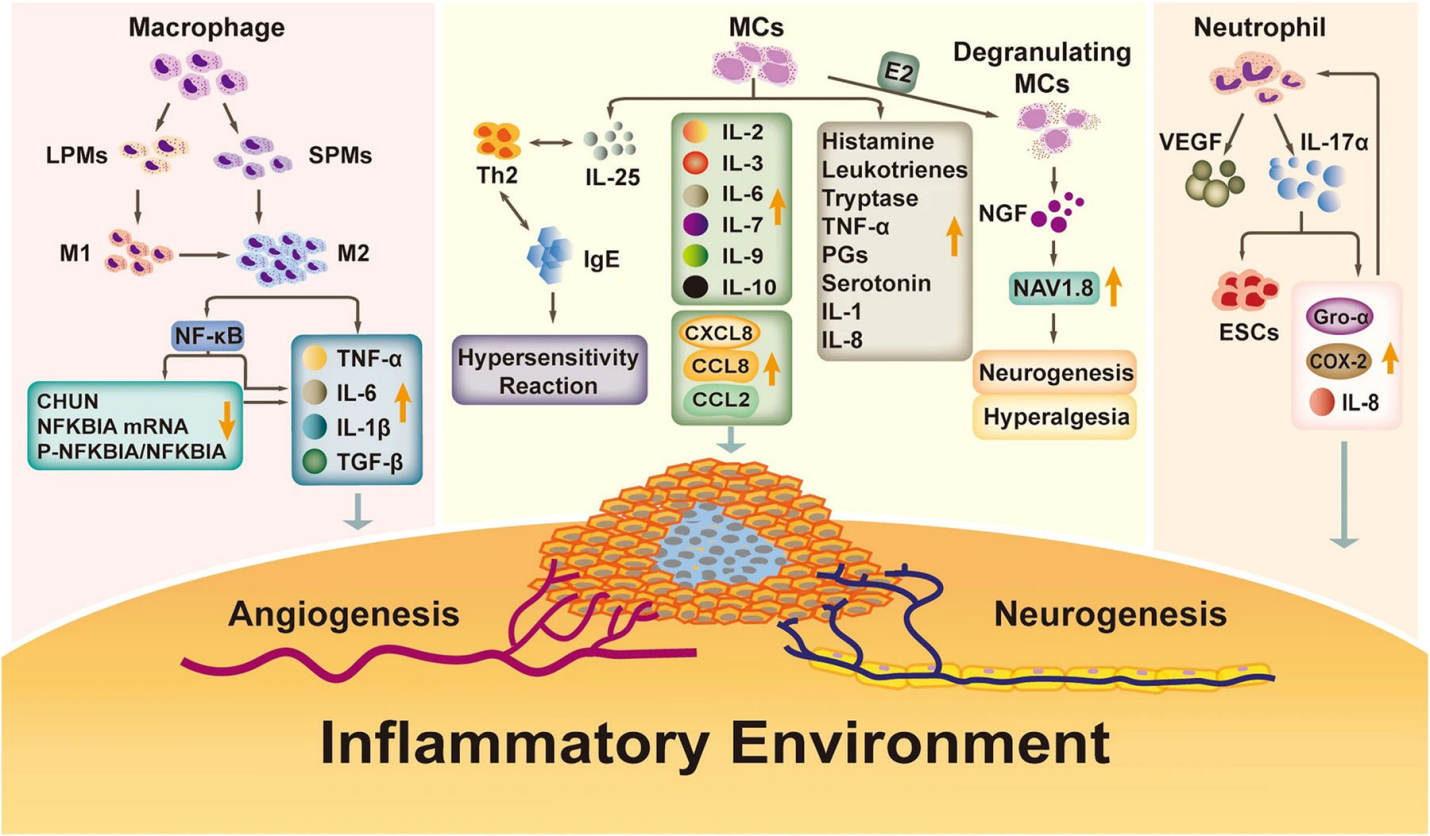
This figure shows how inflammation is involved in the pathogenesis of endometriosis and could induce angiogenesis. This is a reuse of an original figure under Creative Commons Attribution 4.0 International License belongs to the following citation. Wei Y, Liang Y, Lin H, Dai Y, Yao S. Autonomic nervous system and inflammation interaction in endometriosis‐associated pain. *J Neuroinflammation*. 2020;17(1):80. COX‐2, cyclooxygenase‐2; E2, estradiol; IL, interleukin; LPM, large peritoneal macrophage; MC, Mast cells; NFKBIA, NF‐κB inhibitor alpha; PG, prostaglandin; SPM, small peritoneal macrophage; TGF‐β, transforming growth factor β; Th2, T helper 2; TNF‐α, tumor necrosis factor‐α; VEGF, vascular endothelial growth factor.

Inflammation and angiogenesis have common pathways with each other. Inflamed tissues experience hypoxia which leads to the activation of HIF‐1α.^43^ This in turn, triggers the increased expression of VEGF, VEGF receptor (VEGFR), platelet‐derived growth factor (PDGF), stromal cell‐derived factor (SDF‐1), COX‐2, and IL‐1β that are all among the angiogenic factors.^43^, ^44^, ^45^, ^46^ Interestingly, IL‐1β and COX‐2 are also among the most important players of inflammation.^43^ During hypoxia, different proangiogenic factors could be upregulated via nuclear factor κB (NF‐κB) activation among which are IL‐6, TNF‐α, macrophage inflammatory protein 2 (MIP‐2), intercellular adhesion molecule (ICAM), and vascular cell adhesion molecule (VCAM).^47^ Also, inflammation could end in the release of different pro‐inflammatory cytokines among which is TNF‐α, capable of inducing NF‐κB.^43^ Therefore, angiogenesis and inflammation work so close with each other that inhibition of one could potentially inhibit the other. Figure 1 ^48^ shows the role of inflammation in the pathogenesis of endometriosis and inducing angiogenesis in environment.

## 3 HYPOTHESIS

Since inflammation and angiogenesis are quite crucial in the pathogenesis of endometriosis, cannabidiol could be a potent candidate for treatment of this condition due to its antiangiogenic and anti‐inflammatory potential.

### 3.1 Cannabidiol

Cannabinoids are active compounds of plant named *Cannabis sativa* which has been used in medicine for long.^49^ Today, these compounds are categorized into three main groups according to their origin: (I) endocannabinoids produced in body, (II) plant‐derived cannabinoids or phytocannabinoids originating from the *Cannabis sativa* (exocannabinoids), (III) synthetic cannabinoids synthetized in the laboratories (exocannabinoids).^30^ Although different receptors have been known for cannabinoids and endocannabinoids, they exert many of their main actions through two major groups of cannabinoids receptors of cannabinoid receptor 1 (CB1) and CB2.^50^ CBD is a natural non‐psychoactive cannabinoid with affinity to both CB1 and CB2 receptors.^51^ In Jun 2018, CBD became the first cannabinoid which received approval of the Food and Drug Administration (FDA) of United States for two types of seizures under the brand name of Epidiolex®.^52^

### 3.2 Cannabidiol and inflammation

As mentioned, inflammation is a crucial step in the pathogenesis of endometriosis. Data have revealed that CBD has potent anti‐inflammatory properties. Also, in a carrageenan‐induced inflammation murine model, CBD decreased COX activity as well as PGE_2_ and nitric oxide levels.^53^ The macrophages of mouse treated with lipopolysaccharide (LPS) showed to release lower amounts of TNF‐α after treatment with CBD.^54^ Moreover, similar evidence was observed in a mouse model of endotoxin‐induced uveitis which levels of TNF‐α decreased and infiltration of macrophages was inhibited.^55^ Also, it has been shown that CBD could decrease the levels of IL‐1α, IL‐6, and TNF‐α in the LPS‐treated microglial cells which seems to be achieved through modulating NF‐κB.^56^ In the mitogen‐activated human peripheral blood mononuclear cells (PBMC), CBD decreased IL‐1β and TNF‐α.^57^ Also, in the experimental rat models for asthma, CBD has been shown to alleviate inflammation via lowering the levels of TNF‐α, IL‐4, IL‐5, IL‐6, and IL‐13.^58^, ^59^

### 3.3 Cannabidiol and angiogenesis

**Figure 2:**
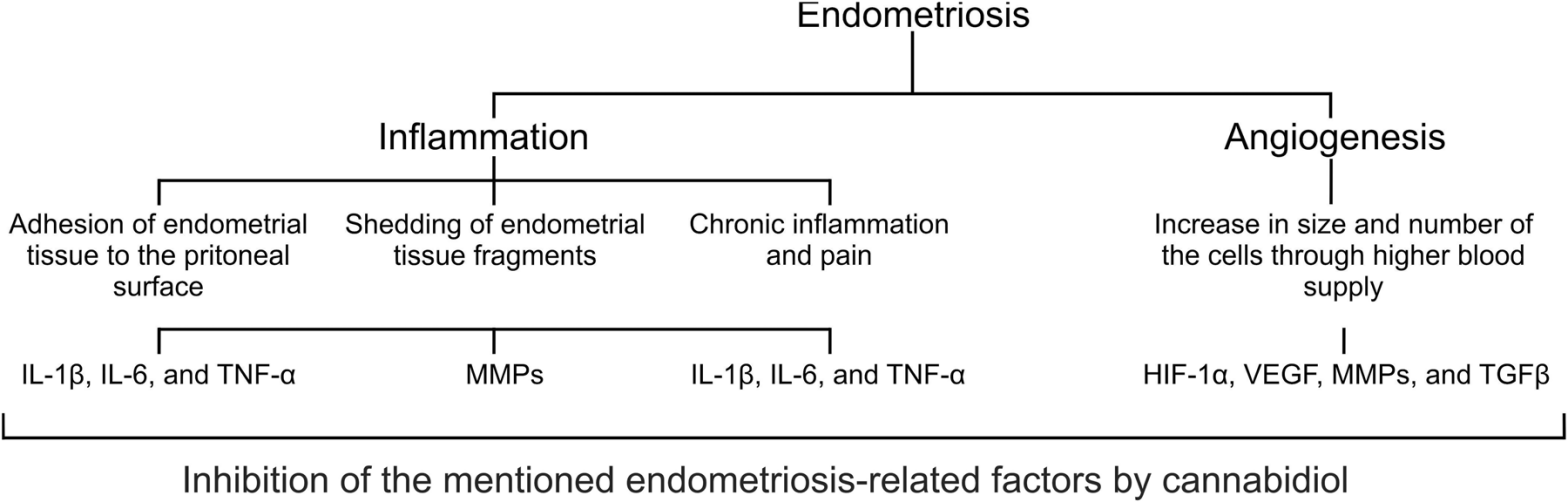
A schematic theme of the role of angiogenesis and inflammation in endometriosis that could be affected by cannabidiol. HIF‐1α, hypoxia‐inducible factor 1α; IL, interleukin; MMP, matrix metalloproteinase; TGF‐β, transforming growth factor β; TNF‐α, tumor necrosis factor‐α; VEGF, vascular endothelial growth factor.

CBD is proven capable of decreasing endothelial cells proliferation and migration (with no toxication and apoptosis induction). These two cell processes are both crucial for angiogenesis. This effect is known to be related to the anti‐tube formation activity of CBD on endothelial cells which is very essential for angiogenesis. These results were followed by inhibition of angiogenesis in vivo in the presence of a strong angiogenic cocktail: VEGF, TNF‐α and heparin. Interestingly, CBD decreases the expression of different proangiogenic factors such as MMP‐2, MMP‐9, TIMP‐1, CXCL16, endothelin 1, IL‐8, and PDGF‐AA.^60^ Also, cannabidiol hydroxyquinone (HU‐331) is able to inhibit angiogenesis in an *ex vivo* model of rat aortic ring (VEGF induced). Moreover, in a mouse tumor‐angiogenesis models, HU‐331 showed potential of decreasing vessels total area, density, and size.^61^ Another study on human endothelial cells demonstrated that CBD decreased high glucose‐induced expression and levels of ICAM‐1 and VCAM‐1 as well as monocytes ability to adhere to the endothelial cells. Moreover, it has been stated that this cannabinoid could decrease the levels of NF‐κB.^62^ As it is shown in Figure 2, both inflammation and angiogenesis are involved in the pathogenesis of endometriosis through different molecular pathways that could be inhibited by CBD.

## 4 CONCLUSION

**Figure 3:**
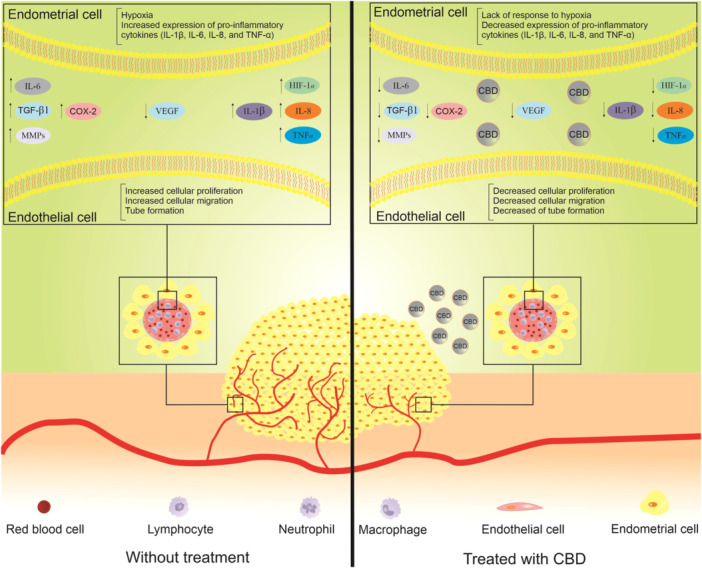
This figure shows which molecules are involved in the pathogenesis of endometriosis which could be targeted by cannabidiol. The left side is the endometriosis condition without treatment and the right side is the same situation with treatment of cannabidiol. The treatment could reduce the number of endometrial cells via inhibition of angiogenesis and inflammation. CBD, cannabidiol; HIF‐1α, hypoxia‐inducible factor 1α; IL, interleukin; MMP, matrix metalloproteinase; TGF‐β, transforming growth factor β; TNF‐α, tumor necrosis factor‐α; VEGF, vascular endothelial growth factor.

So far, the exact mechanism of endometriosis is not fully understood. However, this phenomenon is known as a chronic inflammatory condition accompanied with angiogenesis. Herein, we reviewed the inflammatory aspects of this disease with an eye on angiogenesis. As we showed, the molecular pathways of angiogenesis and inflammation have a lot in common. Thus, it seems that targeting inflammation could be considered hitting two birds with one stone. Considering the potent anti‐inflammatory and antiangiogenic effects of CBD under various other conditions, authors suggest in vitro and in vivo studies on CBD and endometriosis (Figure 3).

## AUTHOR CONTRIBUTIONS

**Roghayeh Anvari Aliabad**: Conceptualization; data curation; writing—original draft; writing—review and editing. **Kamyab Hassanpour**: Data curation; writing—original draft; writing—review and editing. **Amir Hossein Norooznezhad**: Conceptualization; data curation; writing—original draft; writing—review and editing.

## CONFLICT OF INTEREST STATEMENT

The authors declare no conflict of interest.

## Data Availability

All data have been used in the article.
